# Serum Level of 4-Hydroxynonenal in Community-Acquired Pneumonia: A Potential Biomarker for Severity and Prognosis

**DOI:** 10.3389/fmed.2022.798343

**Published:** 2022-06-17

**Authors:** Ya-Lin Jiang, Hong-Yan Liu, Min-Min Tang, Jia-Yi Cheng, Hui Zhao, Lin Fu

**Affiliations:** ^1^Department of Respiratory and Critical Care Medicine, Bozhou People’s Hospital of Anhui Medical University, Bozhou, China; ^2^Department of Respiratory and Critical Care Medicine, Second Affiliated Hospital of Anhui Medical University, Hefei, China

**Keywords:** 4-hydroxynonenal, community-acquired pneumonia, oxidative stress, severity, prognosis

## Abstract

**Background:**

Four-hydroxynonenal (4-HNE) exerts a central role in the pathophysiological process of pulmonary diseases. The aim of this project was to evaluate the correlations between serum 4-HNE with severity and prognosis in patients with community-acquired pneumonia (CAP) by a prospective cohort study.

**Materials and Methods:**

A total of 239 patients with CAP and healthy volunteers were recruited. Fasting blood was collected. Serum 4-HNE was measured with ELISA. Clinical characteristics and demographic information were obtained. The relationships between serum 4-HNE and clinical characteristics were evaluated through the Spearman or Pearson correlation coefficient. The associations of serum 4-HNE with severity and prognosis were estimated through logistic regression analysis.

**Results:**

On admission, serum 4-HNE was upregulated in patients with CAP compared with healthy volunteers. Serum 4-HNE was gradually increased in line with CAP scores. Additionally, elderly patients with CAP were more prone to suffer from 4-HNE elevation. Moreover, serum 4-HNE was positively correlated with CAP severity scores. Meanwhile, the poor prognostic outcomes were tracked among patients with CAP. Higher serum 4-HNE on admission increased the risks of mechanical ventilation, vasoactive agent usage, and death in patients with CAP during hospitalization. The predictive powers for severity and death were increased in serum 4-HNE compared with CAP severity scores and inflammatory cytokines.

**Conclusion:**

Serum 4-HNE on admission is positively correlated with the severity and poor prognosis among patients with CAP, indicating that 4-HNE participates in the pathophysiology of CAP. Serum 4-HNE may be used as an earlier biomarker for diagnosis and prognosis in patients with CAP.

## Background

Community-acquired pneumonia (CAP) is one of the most pervasive fatal infectious diseases. Epidemiological studies revealed that CAP has caused three million deaths across the world, one million deaths in Asia, and six hundred thousand hospitalizations of geriatric patients in the United States every year (1–4). Pneumococcal was one of the most important causes of CAP in children under 5 years of age and adults aged more than 60 years in developed countries (5, 6). CAP is not only related to high mortality but also a major cause of hospitalization (7, 8). Moreover, CAP increases a huge financial burden for the individual family and the whole community (9). Therefore, CAP has attracted more and more attention and become a vital public health problem.

It is widely realized that the excessive production of reactive oxygen species (ROS) can evoke oxidative stress and cellular damage (10, 11). ROS can react with cellular lipids and initiate lipid peroxidation. Polyunsaturated fatty acids (PUFAs), consisting of linoleic acid, arachidonic acid, and docosahexaenoic acid, are an important target for ROS attacks because of the presence of cellular membrane phospholipids (12). 4-Hydroxynonenal (4-HNE) is one of the important products of endogenous lipid peroxidation (10, 13). 4-HNE, above physiological concentration, is a potent alkylating agent, which can react with DNA and proteins, and then generates a wide variety of adducts. Finally, the adducts contribute to evoking cellular stress responses and mutations, destroying cell structures, and disrupting cellular metabolism (14, 15). Previous studies have revealed that 4-HNE is generated and increased in the mice models of lipopolysaccharide-induced acute lung injury and cigarette smoke-evoked emphysema (16, 17). Moreover, a population epidemiological study indicated that 4-HNE is increased in the lungs of patients with chronic obstructive pulmonary disease (COPD) (18). These results suggested that 4-HNE involves in the pathophysiological process of inflammatory lung diseases.

However, the function of 4-HNE in patients with CAP is unclear at present. In addition, the relationships of serum 4-HNE with severity scores and prognostic outcomes were not uncertain in patients with CAP. We conjectured that 4-HNE may exert a significant role in the process of CAP. Therefore, serum 4-HNE was detected in the different graded patients. The relationships of serum 4-HNE with the severity and prognosis were evaluated in patients with CAP through a prospective cohort study.

## Materials and Methods

### Study Design and Subjects

**FIGURE 1:**
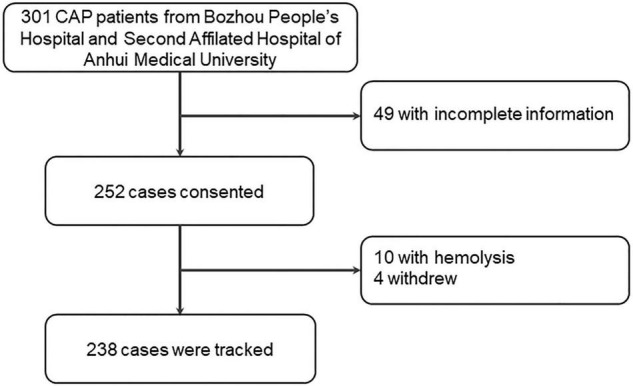
Flow diagram of recruitment and follow-up research in the cohort study.

All patients with CAP were recruited from Bozhou People’s Hospital and the Second Affiliated Hospital of Anhui Medical University. This project began from June 2019 to March 2021. All the patients have not been admitted to a hospital over the past half-year. All eligible subjects must meet CAP diagnostic criteria: (I) a new patchy infiltrate, leaf or segment consolidation, ground-glass opacity, or interstitial change were observed through a chest radiograph; (II) all patients had CAP in the community rather than in the hospital; and (III) at least one of the following signs was observed in patients: coughing, sputum production, and dyspnea occurred; core body temperature was higher than 38.0°C; abnormal breath sounds and rales were observed on auscultation; and the number of white blood cells (WBCs) was greater than 10 × 10^9^ L or less than 4 × 10^9^L (19). The inclusion criteria were as follows: (I) all subjects were diagnosed as patients with CAP; (II) positive blood or sputum cultures were observed; (III) all participators were more than 18 years of age; and (IV) all subjects volunteered to take part in the follow-up treatment and research (20–22). Additionally, exclusion criteria for participants were as follows: (I) pregnant women; (II) participants less than 18 years of age; (III) patients with lung carcinoma, tuberculosis, or chronic airway diseases; (IV) patients undergoing xenotransplantation or bone marrow transplantation; and (V) immunosuppressed patients. At first, 301 subjects who confirmed with CAP were rerolled and 49 patients without complete information were excluded. In addition, four patients declined to participate and ten serum samples had hemolysis. Finally, a total of 238 patients with CAP were included in this study (Figure 1). In addition, age- and sex-matched healthy volunteers were enrolled in the physical examination center of the Second Affiliated Hospital of Anhui Medical University. All healthy volunteers did not have other pulmonary diseases or basic diseases. Finally, 238 control subjects were recruited for this study. Simultaneously, fasting samples were collected among all participants at 6:30 a.m. on admission. Meanwhile, demographic information and laboratory results were obtained. The severity of patients with CAP was accessed with CAP severity scores, such as the Pneumonia Severity Index (PSI), CRB-65, SMART-COP, Acute Physiology and Chronic Health Evaluation (APACHE) II, CURXO, and CURB-65. The prognostic outcomes were tracked in patients with CAP. During hospitalization, the exacerbation of CAP was defined by the following conditions: intensive care unit (ICU) admission; receiving mechanical ventilation or vasoactive agent use; and death. The Biomedicine Ethics Committees of Bozhou People’s Hospital and the Second Affiliated Hospital of Anhui Medical University supported this project. This research was in accordance with the Declaration of Helsinki. All participators offered informed consent.

### Enzyme-Linked Immunosorbent Assay

Fasting blood samples were collected before treatment within 24 h after hospitalization. Then, blood samples were centrifuged at 3,500 *g*/min and serum samples were obtained (23, 24). All serum samples were stored in a super cold refrigerator spare. The concentration of 4-HNE was quantitated *via* enzyme-linked immunosorbent assay (ELISA) as we have described previously (25, 26). The 4-HNE kits were bought from Cusabio, Wuhan, China.^1^

### Statistical Analysis

Statistical analyses were conducted *via* SPSS software. Continuous data were expressed as median or mean. Categorical data were expressed as frequency. To compare the differences between the two groups, continuous data were analyzed using an independent sample *t*-test, and categorical data were analyzed using the chi-square test. The relationships between serum 4-HNE and clinical indices were evaluated using the Spearman or Pearson correlation coefficient. The relationships between serum 4-HNE with severity and prognosis were analyzed using linear or logistic regression. The predictive power was analyzed using the receiver operating characteristic (ROC) area under the curve (AUC). Statistical significance was regarded as *p* < 0.05.

## Results

### Baseline Information

In this study, baseline information was compared and analyzed. As shown in Table 1, we found that no significant difference in age, gender, and smoking was observed between the CAP group and the control group. In addition, a routine blood test was carried out among all subjects. The data indicated that the numbers of WBCs, neutrophils, and basophils rose in the CAP group. On the contrary, the count of lymphocytes was decreased in patients with CAP. In addition, alanine aminotransferase (ALT) was increased in patients with CAP. Furthermore, the levels of inflammatory cytokines, C-reactive protein (CRP), and interleukin-6 (IL-6) were higher in patients with CAP compared with control subjects (Table 1).

**TABLE 1:** Demographic characteristics and clinical data.

Variables	CAP (*n* = 238)	Control (*n* = 238)	*P*
Age (years)	62.05 ± 2.15	62.5 ± 2.16	0.351
Male, *n* (%)	143 (60.1)	154 (64.7)	0.298
BMI	22.55 ± 0.43	N.A.	N.A.
Smoker, *n* (%)	43 (18.1)	55 (23.1)	0.174
Systolic pressure (mmHg)	125.87 ± 2.36	119.61 ± 3.35	0.215
Diastolic pressure (mmHg)	76.36 ± 0.89	74.65 ± 1.12	0.512
White blood cell (10^9^/L)	8.36 ± 0.37	5.56 ± 0.43	<0.01
Neutrophil (10^9^/L)	7.89 ± 0.86	3.12 ± 0.31	<0.01
Lymphocyte (10^9^/L)	2.00 ± 0.51	2.11 ± 0.21	0.365
Monocyte (10^9^/L)	0.63 ± 0.051	0.37 ± 0.031	0.040
Eosinophil (10^9^/L)	0.11 ± 0.016	0.12 ± 0.027	0.165
Basophil (10^9^/L)	0.17 ± 0.077	0.018 ± 0.054	<0.01
C-reactive protein (mg/L)	86.71 ± 6.38	33.61 ± 7.65	<0.01
Interleukin-6 (pg/mL)	113.38 ± 4.62	42.32 ± 5.24	<0.01

*All data were expressed mean ± SEM.*

*N.A., not available. BMI, body mass index.*

### The Levels of Serum 4-Hydroxynonenal in Patients With Community-Acquired Pneumonia and Control Subjects

**FIGURE 2:**
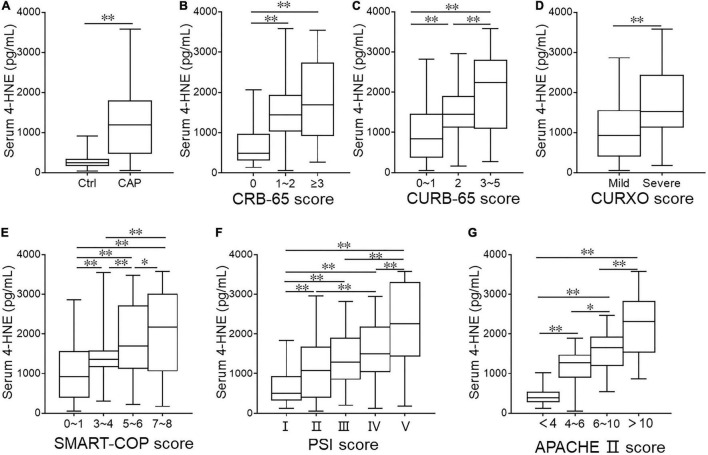
The levels of serum 4-hydroxynonenal (4-HNE) in patients with community-acquired pneumonia (CAP) and healthy volunteers. **(A)** Serum 4-HNE was determined in patients with CAP and healthy volunteers through ELISA. **(B–G)** The levels of serum 4-HNE were measured in CAP patients with different severity scores through ELISA. **(B)** CRB-65 score. **(C)** CURB-65 score. **(D)** CURXO score. **(E)** SMART-COP score. **(F)** Pneumonia severity index (PSI) score. **(G)** Acute physiology and chronic health evaluation (APACHE) II score. *p < 0.05 and ^**^p < 0.01.

Serum 4-HNE was determined among all participators through ELISA. As shown in Figure 2A, we found an enhanced level of 4-HNE in patients with CAP than those in control subjects. As shown in Figure 2B, the level of serum 4-HNE was higher in the rank of ≥3 scores than those in the rank of <3 scores in CRB-65 score. According to the CURB-65 score, the level of serum 4-HNE was gradually upregulated in parallel with the severity score (Figure 2C). In accordance with the CURXO score, the level of serum 4-HNE was remarkedly increased in severe patients with CAP (Figure 2D). Meanwhile, among CAP patients with diverse scores of SMART-COP, PSI, and APACHE II, the levels of serum 4-HNE were further analyzed. We found that the levels of serum 4-HNE were gradually upregulated in line with the scores among patients with CAP (Figures 2E–G).

### The Risk Factors of Serum 4-Hydroxynonenal Elevation on Admission Among Patients With Community-Acquired Pneumonia

The effects of demographic characteristics on serum 4-HNE elevation were estimated among patients with CAP. Although univariate logistic regression suggested that no effect of male, body mass index (BMI), systolic pressure, diastolic pressure, diabetes, cerebral infarction, hypertension, bronchitis, and coronary heart disease on serum 4-HNE levels among patients with CAP was observed. The odds ratio [*OR*] of age was 1.057 (95% *CI*: 1.037, 1.077), the *OR* of being a smoker was 1.102 (95% *CI*: 1.005, 1.456), and the *OR* of systolic pressure was 1.016 (95% *CI*: 1.002, 1.029) among patients with CAP (Table 2). Although there was no obvious effect of smoking and systolic pressure on serum 4-HNE elevation on admission in the multivariate logistic regression model, age increased the risk of serum 4-HNE elevation among patients with CAP (*OR* = 1.087; 95% *CI*: 1.044, 1.389) (Table 2).

**TABLE 2:** The risk factors of 4-hydroxynonenal (4-HNE) elevation in community-acquired pneumonia (CAP) patients.

Variables	Univariate		Multivariate	
		
	OR (95% CI)	*P*	OR (95% CI)	*P*
Male	1.250 (0.745, 2.098)	0.306	0.605 (0.297, 1.365)	0.236
Age	1.057 (1.037, 1.077)	**<0.001**	**1.087 (1.044, 1.389)**	**<0.001**
BMI	0.949 (0.874, 1.030)	0.211	0.947 (0.878, 1.078)	0.289
Smoker	**1.102 (1.005, 1.456)**	**0.011**	1.085 (0.965, 1.345)	0.087
Systolic pressure	**1.016 (1.002, 1.029)**	**0.026**	1.023 (0.979, 1.066)	0.314
Diastolic pressure	0.994 (0.973, 1.015)	0.551	0.990 (0.951, 1.044)	0.602
Hypertension	0.643 (0.361, 1.147)	0.135	1.128 (0.415, 2.874)	0.654
Diabetes	0.536 (0.216, 1.329)	0.178	0.723 (0.257, 2.547)	0.745
Cerebral infarction	0.796 (0.317, 1.997)	0.627	6.541 (0.265, 106.354)	0.125
Acute bronchitis	1.778 (0.675, 4.685)	0.244	2.122 (0.874, 8.587)	0.201
Coronary heart disease	0.552 (0.157, 1.937)	0.353	0.657 (0.165, 7.854)	0.775

*Bold values indicated statistical significance.*

### Relationships Between Serum 4-Hydroxynonenal and Physiological Hallmarks in Patients With Community-Acquired Pneumonia

The relationships between serum 4-HNE and routine blood indicators were evaluated among patients with CAP. As shown in Table 3, serum 4-HNE was significantly and inversely related to lymphocytes. Besides, the associations among serum 4-HNE with renal function, liver function, and myocardial function were estimated. As shown in Table 3, the level of serum 4-HNE was positively correlated with ALT, aspartate aminotransferase (AST), urea nitrogen, creatinine, α-Hydroxybutyrate dehydrogenaseα (α-HBDH), and lactate dehydrogenase (LDH) in patients with CAP. Finally, the relationships between 4-HNE and inflammatory markers were explored. We found that serum 4-HNE was positively correlated with D-Dimer, CRP, and IL-6 among patients with CAP. Additionally, there was a weakly negative correlation between serum 4-HNE and platelet (PLT) in CAP cases (Table 3).

**TABLE 3:** Associations between serum 4-hydroxynonenal (4-HNE) and clinical characteristics in community-acquired pneumonia (CAP) patients.

Variables	WBC	Neutrophil	Lymphocyte	Basophil	ALT
*r*	0.115	–0.063	–0.185	–0.107	0.188
*P*	0.075	0.332	0.004	0.100	0.004

**Variables**	**AST**	**Uric acid**	**Urea nitrogen**	**Creatinine**	**α -HBDH**

*r*	0.201	0.105	0.145	0.162	0.204
*P*	0.002	0.105	0.026	0.012	0.009

**Variables**	**CK**	**CKMB**	**LDH**	**cTnI**	**Mb**

*r*	0.029	0.097	0.215	0.090	0.113
*P*	0.719	0.219	0.007	0.316	0.210

**Variables**	**D-Dimer**	**PLT**	**PCT**	**IL-6**	**CRP**

*r*	0.134	–0.134	0.114	0.354	0.498
*P*	0.040	0.039	0.144	0.005	<0.001

*ALT, alanine aminotransferase; AST, aspartate aminotransferase; α-HBDH, α-hydroxybutyrate dehydrogenaseα; CK, creatine kinase; CKMB, creatine kinase isoenzyme; LDH, lactate dehydrogenase; cTnI, cardiac troponin I; Mb, myoglobin; PLT, platelet; PCT, procalcitonin; IL-6, interleukin-6; CRP, C-reactive protein.*

### Relationships Between Serum 4-Hydroxynonenal and Severity Scores in Patients With Community-Acquired Pneumonia

The relationships between serum 4-HNE and CAP severity scores were analyzed through linear regression. Age was adjusted in the different regression models. We found that serum 4-HNE was positively related to CURB-65, CRB-65, CURXO (severe), SMART-COP, and APACHE II in patients with CAP (Table 4). For the sake of confirming the associations between serum 4-HNE and severity scores, a logistic regression analysis was conducted. The levels of serum 4-HNE were divided into trisection among patients with CAP by tertiles, such as low (Tertile 1, <680.2 pg/ml), medium (Tertile 2, 680.2–1,483.4 pg/ml), and high (Tertile 3, >1,483.4 pg/ml) grades. Compared with CAP patients from the lowest 4-HNE group, those from the highest 4-HNE group had an increase of 6.857 scores, 4.919 scores, 2.512 scores, 4.509 scores, 3.223 scores, and 16.654 scores in CURB-65, CRB-65, PSI, CURXO, SMART-COP, and APACHE II, respectively (Table 4).

**TABLE 4:** Associations between serum 4-hydroxynonenal (4-HNE) and community-acquired pneumonia (CAP) severity scores.

Variables	Estimated changes continues serum 4-HNE	Estimated changes (95% CI) by tertiles of serum 4-HNE			*P* trend
		
		Low (<680.2 pg/mL)	Medium (680.2∼1483.4 pg/mL)	High (>1483.4 pg/mL)	
N	239	117	116	117	
CURB-65	1.021 (1.005, 1.122)**	0 (Ref)	3.876 (1.705, 8.814)**	6.857 (2.919, 16.111)**	**<0.001**
CRB-65	1.035 (1.007, 1.154)*	0 (Ref)	3.525 (1.446, 8.592)**	4.919 (1.976, 12.241)**	**0.008**
PSI	1.628 (0.874, 3.030)	0 (Ref)	1.314 (0.627, 2.754)	**2.512 (1.144, 5.516)** *	0.068
CURXO (Severe)	1.085 (1.006, 1.155)**	0 (Ref)	3.291 (1.347, 8.038)**	4.509 (1.799, 11.306)**	**<0.001**
SMART-COP	1.108 (1.045, 1.748)**	0 (Ref)	**2.646 (1.262, 5.549)** *	3.223 (1.480, 7.017)**	**0.005**
APACHE II	1.101 (1.025, 1.879)**	0 (Ref)	5.324 (2.354, 16.654)**	16.654 (3.678, 54.874)**	**<0.001**

*Models were adjusted for age. CI, confidence interval.*

*Bold values indicated statistical significance.*

*p < 0.05 and **p < 0.01.

### Higher Serum 4-Hydroxynonenal on Admission Increased the Risk of Adverse Prognosis Among Patients With Community-Acquired Pneumonia

The effect of 4-HNE elevation on admission on the prognosis was evaluated in patients with CAP using logistic regression. As expressed in Table 5, univariate logistic regression found that a higher serum 4-HNE on admission increased the risks of mechanical ventilation, vasoactive agent usage, ICU admission, longer hospital stays, and death during hospitalization. Then, age was adjusted in the multivariable logistic regression model. The results found that patients with CAP in the highest 4-HNE grade had an elevation of 2.495 times, 3.923 times, and 14.561 times in mechanical ventilation, vasoactive agent usage, and death than those in the lowest 4-HNE grade during hospitalization (Table 5).

**TABLE 5:** The associations between serum 4-hydroxynonenal (4-HNE) and prognostic outcomes in community-acquired pneumonia (CAP) patients.

4-HNE	Prognosis	Univariable		Multivariable*	
			
		OR (95% CI)	*P*	OR (95% CI)	*P*
Low	Mechanical ventilation	1	—	1	—
Medium		**2.576 (1.157, 5.736)**	**0.020**	1.963 (0.852, 4.520)	0.113
High		**4.182 (1.921, 9.102)**	**<0.001**	**2.495 (1.059, 5.878)**	**0.037**
Low	Vasoactive agent	1	—	1	—
Medium		3.074 (0.935, 10.105)	0.064	2.395 (0.706, 8.122)	0.161
High		**5.918 (1.912, 18.313)**	**0.002**	**3.923 (1.158, 13.291)**	**0.028**
Low	ICU admission	1	—	1	—
Medium		**2.386 (1.116, 5.102)**	**0.025**	1.768 (0.798, 3.917)	0.123
High		**3.436 (1.633, 7.227)**	**0.001**	1.914 (0.839, 4.366)	0.160
Low	Hospital stays	1	—	1	—
Medium		1.927 (0.961, 3.864)	0.065	1.445 (0.649, 3.217)	0.368
High		**2.107 (1.043, 4.258)**	**0.038**	1.651 (0.796, 3.425)	0.178
Low	Death	1	—	1	—
Medium		6.493 (0.763, 55.231)	0.087	5.271 (0.608, 45.696)	0.131
High		**18.231 (2.345, 141.711)**	**0.006**	**14.561 (1.721, 123.206)**	**0.014**

*Age was adjusted. OR, odd ratio.

*Bold values indicated statistical significance.*

### The Predictive Powers for Severity and Death

**FIGURE 3:**
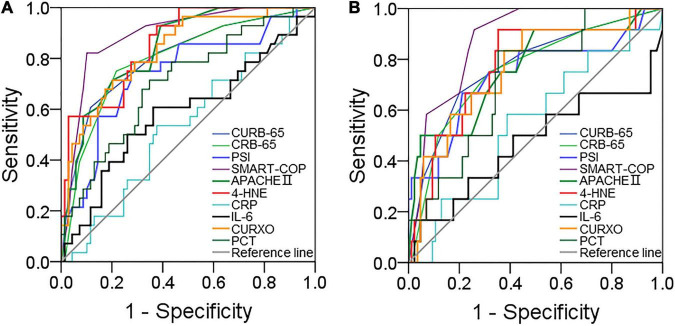
The predictive powers for severity and death. The predictive powers for severity and death were analyzed by the receiver operating characteristic (ROC) curve between serum 4-HNE with CAP severity scores and inflammatory cytokines among patients with CAP. **(A)** The predictive power for severity was estimated. **(B)** The predictive power for death was estimated.

The predictive powers for severity were estimated between serum 4-HNE and different clinical characteristics among patients with CAP through the ROC AUC. As shown in Figure 3A, the AUCs of severity were as follows: SMART-COP, 0.893; serum 4-HNE, 0.856; APACHE II, 0.840; CURXO, 0.832; CRUB-65, 0.805; CRB-65, 0.795; PSI, 0.742; PCT, 0.705; CRP, 0.856; and IL-6, 0.536. The level of serum 4-HNE >1,063.7 pg/ml had 92.9% sensitivity and 78.6% specificity for indicating severity. Moreover, the predictive capacities for death were assessed. As shown in Figure 3B, the AUCs of death were as follows: SMART-COP, 0.877; serum 4-HNE, 0.848; CRUB-65, 0.774; APACHE II, 0.765; CURXO, 0.762; PSI, 0.736; CRB-65, 0.758; PCT, 0.719; CRP, 0.542; and IL-6, 0.471. The optimal cutoff concentration of serum 4-HNE for death was 1,308.5 pg/ml. The specificity was 91.7%, and the sensitivity was 79.5%.

## Discussion

This project estimated the relationships of serum 4-HNE with severity and prognosis among patients with CAP. This project mainly revealed that: (1) serum 4-HNE was upregulated in patients with CAP in comparison with healthy participants; (2) serum 4-HNE gradually increased in line with CAP scores; (3) elderly patients with CAP were more susceptible to suffering from serum 4-HNE elevation; (4) serum 4-HNE was positively associated with CAP severity scores; (5) higher serum 4-HNE at the early stage increased the risk of mechanical ventilation, vasoactive agent usage, and death during hospitalization; and (6) predictive capacities for severity and death were higher in serum 4-HNE compared with known CAP severity scores and inflammatory cytokines.

Four-hydroxynonenal is one reactive breakdown product of the lipid peroxides (10, 13). Previous studies have revealed that the expressions of 4-HNE are significantly increased in the lungs of mice with acute lung injury or emphysema (16, 17). Not only that, the levels of pulmonary 4-HNE were upregulated in patients with COPD (18). In addition, the autopsy report revealed that 4-HNE is more highly expressed in the lungs and kidneys of patients with COVID-19 (27, 28). However, the role of 4-HNE was unclear in patients with CAP. Some studies from our team have demonstrated that inflammation and oxidative stress exert important roles in the pathophysiology of CAP (29–32). Hence, it is reasonable to infer that 4-HNE may be involved in the process of CAP. Serum 4-HNE was increased among patients with CAP. In addition, the level of serum 4-HNE was gradually upregulated in parallel with CAP scores. The previous study demonstrated that very old adults are prone to suffer from critical pneumonia (32). In the current research, the effects of demographic characteristics on serum 4-HNE were estimated among patients with CAP. Our results found that older patients were more susceptible to suffering from serum 4-HNE elevation among patients with CAP. These results suggested that 4-HNE is involved in the pathophysiology progress of CAP. Age is one independent risk factor of serum 4-HNE elevation among patients with CAP.

Mounting data have demonstrated that multiple organ injury is observed in the process of CAP (33, 34). In this article, the associations of serum 4-HNE and characteristics of organ function were analyzed. The results indicated that serum 4-HNE was associated with several clinical characteristics among patients with CAP. For example, the level of serum 4-HNE was strongly correlated with the parameters of routine blood, renal function, liver function, and myocardial function in subjects with CAP. In addition, inflammation always plays an important role in pneumonia and many inflammatory cytokines are increased in patients with pneumonia (35–38). Increasing data found that the status of oxidative stress can predict the severity of CAP (39, 40). Consequently, the relationships between serum 4-HNE and CAP severity scores were assessed. Linear and logistic regression analysis stated that the level of serum 4-HNE was positively associated with CRB-65, CURB-65, PSI, SMART-COP, APACHE II, and CURXO scores in patients with CAP. These results provided evidence that serum 4-HNE is positively related to CAP severity scores.

Former studies have demonstrated that the markers of oxidative stress have the predictive capacity of diseases’ prognosis. Excessive oxidative stress always indicates a bad prognosis in patients with cerebral malaria (41). 8-Isoprostane, a biomarker of lipid peroxidation in the process of oxidative damage, is positively associated with the death risk in patients with CAP (32). Malondialdehyde, the product of lipid peroxidation, is positively correlated with poor prognosis among patients with chronic heart failure (42). The higher level of ROS, which is the product of the reaction of nitric oxide and superoxide, increases the risks of death and ICU admission within 30 days for patients with CAP (43). Thus, we evaluated the relationship between serum 4-HNE and the prognosis among patients with CAP. The results clarified that serum 4-HNE on admission was positively associated with the risks of mechanical ventilation, vasoactive agent usage, and death in patients with CAP. These results suggest that serum 4-HNE on admission is positively related to poor prognostic outcomes among patients with CAP.

A ROC curve was analyzed to explore the application of detecting serum 4-HNE in CAP management. The results found that though there were similar predictive powers for severity between serum 4-HNE and CAP severity scores, the predictive capacities for severity and death were significantly increased compared with inflammatory cytokines among patients with CAP. In addition, the predictive capacities for death were higher in serum 4-HNE than those in many known CAP severity scores. Therefore, serum 4-HNE may be regarded as a diagnostic and prognostic biomarker for severity and prognosis in patients with CAP. On admission, when the concentration of serum 4-HNE was higher than 1,063.7 pg/ml, the risk of critically ill patients with CAP was remarkedly increased. Additionally, once the content of serum 4-HNE was more than 1,308.5 pg/ml, the risk of death was significantly increased during hospitalization. Consequently, serum 4-HNE detection can indicate partial CAP patients with higher serum 4-HNE need more medical attention, earlier therapy, and intervention. It is helpful to reduce the death risk of critically ill patients with CAP.

This study mainly assessed the relationships between serum 4-HNE on admission with severity and prognosis among patients with CAP. This research enhanced the understanding of 4-HNE in CAP. Nevertheless, there are several defects in this study. First, this research just had a modest sample size from one hospital in China. So, the generalizability of these conclusions may be limited. Second, only the circulatory level of 4-HNE was measured in patients with CAP. The local expression of 4-HNE in the lungs was unclear. Maybe, the level of pulmonary 4-HNE is more convincing and better in patients with CAP. Third, this was a longitudinal study. Therefore, the mechanism of 4-HNE elevation remained unknown in patients with CAP. *In vivo* and *in vitro* studies are favorable to clear up this confusion in patients with CAP.

## Conclusion

Taken together, this research mainly found that serum 4-HNE is upregulated in patients with CAP. Elderly patients with CAP are more prone to suffer from 4-HNE elevation in serum. Serum 4-HNE is positively related to severity and poor prognosis among patients with CAP. The level of serum 4-HNE on admission has stronger predictive capacities for severity and death than those in many known CAP severity scores and inflammatory cytokines, demonstrating that 4-HNE exerts important roles in the pathophysiology of CAP. Our results provide evidence that serum 4-HNE may be used as an early biomarker for diagnosis and prognosis among patients with CAP.

## Data Availability Statement

The raw data supporting the conclusions of this article will be made available by the authors, without undue reservation.

## Ethics Statement

The studies involving human participants were reviewed and approved by the Ethics Committee of the Bozhou People’s Hospital (2021-10). The patients/participants provided their written informed consent to participate in this study.

## Author Contributions

LF and HZ contributed to the design of the study, funding support, statistical analyses, and drafting of the manuscript. Y-LJ, M-MT, H-YL, and J-YC contributed to sample collection, data interpretation, recruiting patients, and obtaining their written informed consent. All authors have read carefully and approved the final manuscript.

## Conflict of Interest

The authors declare that the research was conducted in the absence of any commercial or financial relationships that could be construed as a potential conflict of interest. The reviewer C-MF declared a shared affiliation with the authors to the handling editor at the time of review.

## Publisher’s Note

All claims expressed in this article are solely those of the authors and do not necessarily represent those of their affiliated organizations, or those of the publisher, the editors and the reviewers. Any product that may be evaluated in this article, or claim that may be made by its manufacturer, is not guaranteed or endorsed by the publisher.

## Footnotes

1
https://www.cusabio.com/
